# ^125^I Brachytherapy in Locally Advanced Nonsmall Cell Lung Cancer After Progression of Concurrent Radiochemotherapy

**DOI:** 10.1097/MD.0000000000002249

**Published:** 2015-12-11

**Authors:** Zhanwang Xiang, Guohong Li, Zhenyin Liu, Jinhua Huang, Zhihui Zhong, Lin Sun, Chuanxing Li, Funjun Zhang

**Affiliations:** From the State Key Laboratory of Oncology in South China, Collaborative Innovation Center for Cancer Medicine, Sun Yat-sen University Cancer Center (ZX, GL, JH, ZZ, LS, CL, FZ), and Guangzhou Women and Children Health Care Center, Guangzhou, China (ZL).

## Abstract

To investigate the safety and effectiveness of computed tomography (CT)-guided ^125^I seed implantation for locally advanced nonsmall cell lung cancer (NSCLC) after progression of concurrent radiochemotherapy (CCRT).

We reviewed 78 locally advanced NSCLC patients who had each one cycle of first-line CCRT but had progressive disease identified from January 2006 to February 2015 at our institution. A total of 37 patients with 44 lesions received CT-guided percutaneous ^125^I seed implantation and second-line chemotherapy (group A), while 41 with 41 lesions received second-line chemotherapy (group B).

Patients in group A and B received a total of 37 and 41 first cycle of CCRT treatment. The median follow-up was 19 (range 3–36) months. After the second treatment, the total response rate (RR) in tumor response accounted for 63.6% in group A, which was significantly higher than that of group B (41.5%) (*P* = 0.033). The median progression-free survival time (PFST) was 8.00 ± 1.09 months and 5.00 ± 0.64 months in groups A and B (*P* = 0.011). The 1-, 2-, and 3-year overall survival (OS) rates for group A were 56.8%, 16.2%, and 2.7%, respectively. For group B, OS rates were 36.6%, 9.8%, and 2.4%, respectively. The median OS time was 14.00 ± 1.82 months and 10.00 ± 1.37 months for groups A and B, respectively (*P* = 0.059). Similar toxicity reactions were found in both groups. Tumor-related clinical symptoms were significantly reduced and the patients’ quality of life was obviously improved.

CT-guided ^125^I seed implantation proved to be potentially beneficial in treating localized advanced NSCLC; it achieved good local control rates and relieved clinical symptoms without increasing side effects.

## INTRODUCTION

Lung cancer is one of the most commonly occurring malignancies and the leading cause of cancer-related death worldwide, most of which (75%–80%) is nonsmall cell lung cancer (NSCLC).^1^ To our disappointment, approximately 55% of patients who have been newly diagnosed with NSCLC have distant metastases.^2^ Locally advanced, stage III–IV NSCLC is one of the major battlegrounds in clinical treatment and research in lung cancer. Only a minority of patients with stage III–IV lung cancer are actually treated with surgery.^3^ Most NSCLC patients (about 80%) miss the opportunities for surgical resection once they are diagnosed.^4,5^ Therefore, chemotherapy combined with external beam radiotherapy has played a major role in the management of patients with unresectable lung cancer patients.^6,7^ Numerous clinical studies have also confirmed the effectiveness of chemotherapy combined with radiotherapy in the treatment of advanced NSCLC,^8,9^ which could prolong survival time and obviously improve the quality of life of patients.^10^ However, at present, a great number of patients cannot tolerate the currently available treatment modalities mainly owing to their poor general condition,^11^ tumor staging and grading, and severe toxicity after radiotherapy and chemotherapy (myelosuppression, nausea, vomiting, radiation pneumonitis, etc.), especially that affecting important organs and tissues (heart, esophagus, and large blood vessels). Even if using the latest technology, such as sophisticated 3-dimensional computerized planning systems, multileaf beam collimators, or altered fractionation schedules, the detrimental side effects of therapy cannot be avoided. Thus, the external beam radiotherapy dose must be decreased rapidly, which may make the eradication of the local tumor difficult and eventually lead to residual tumor. This is considered one of the important factors of tumor recurrence and metastasis.^12^

To break through the limitation of external radiotherapy and improve the clinical efficacy of tumor treatment of patients with NSCLC, ^125^I brachytherapy was developed. This new modality leads to a more extensive necrosis of the tumor and further improved the quality of life of patients. Previous studies have shown that ^125^I seed implantation is an acceptable and useful minimally invasive therapy for tumors in other organs.^13–15^ In fact, ^125^I seed implantation has been widely used in pancreatic cancer, liver cancer, gynecologic malignancies, and brain cancer.^16–18^ Many studies have also begun to explore the ^125^I seed implantation treatment for malignant lung tumors. The results showed that percutaneous pulmonary ^125^I seed implantation was safe and feasible in lung tissue, it achieved better local tumor control, while not increasing other serious complications.^19^

The ^125^I seed releases low doses of X- and γ-rays continuously. Its half-life is 59.6 days, and the radiation radius is 1.7 cm with a total dose administration of approximately 110 to 160 Gy. The radiation energy of ^125^I seeds decreases rapidly with the increase in distance.^20^ Therefore, computed tomography (CT)-guided ^125^I brachytherapy can target the entire dose irradiation to the local tumor, while it provides a lower dose to normal adjacent tissue. Additionally, the time of local tumor remission is obviously decreased, and there is no increase in the risk of radiation-related toxicity. Brachytherapy combined with concurrent chemotherapy is more effective.

Thus, the purpose of this study was to evaluate the safety and effectiveness of CT-guided ^125^I seed implantation after failure of concurrent radiochemotherapy (CCRT) for locally advanced NSCLC.

## MATERIALS AND METHODS

### Patient Selection

From January 2006 to February 2015, we recruited 78 patients who were diagnosed with locally advanced NSCLC at our hospital, the Sun Yat-Sen University Cancer Center. Each patient underwent a first cycle of CCRT but continued to experience disease progression. Patients were then randomly divided into 2 groups, 37 patients (group A) were transferred to percutaneous ^125^I seed implantation therapy and second-line chemotherapy, and 41 patients (group B) were given second-line chemotherapy. The retrospective study was approved by the institutional review board at our hospital. The characteristics of individual patients and tumors are summarized in Table 1.

**TABLE 1:**
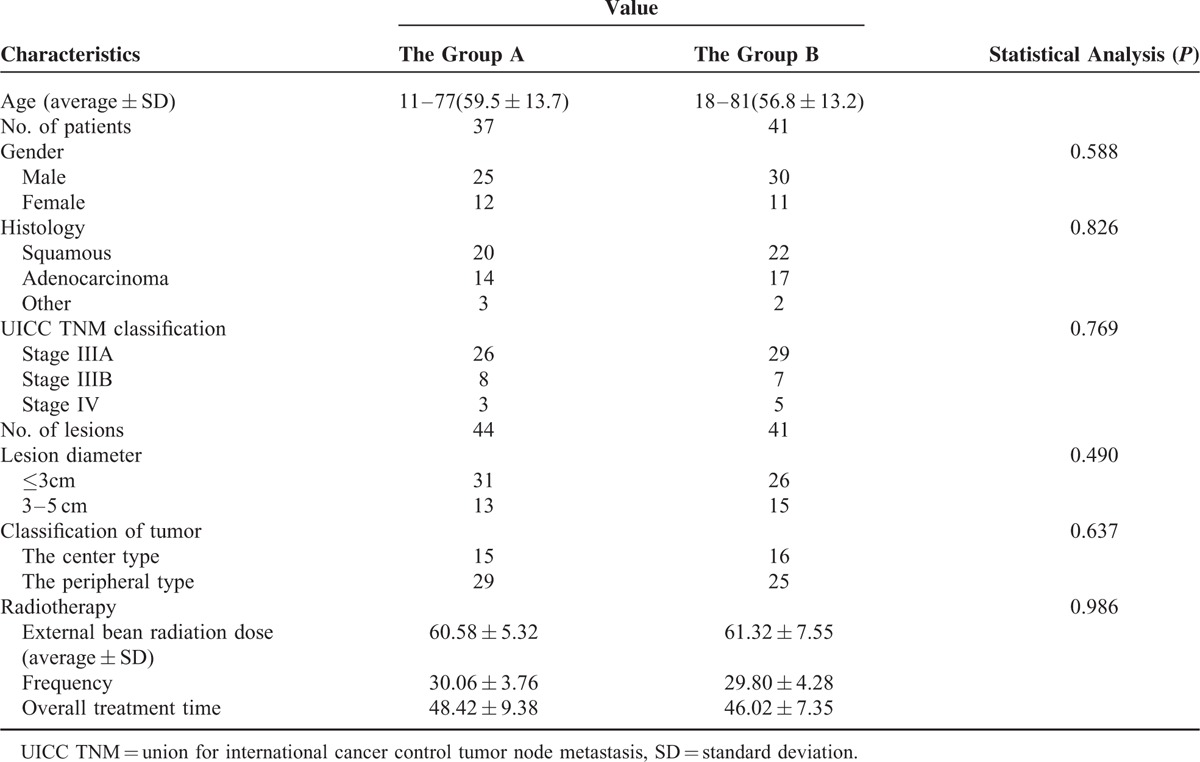
Summary of Patient and Tumor Characteristics

### Inclusion and Exclusion Criteria

Study inclusion criteria were as follows: diagnosis of NSCLC confirmed by biopsy, less than 3 unilateral lung lesions, single lesion with a diameter less than 5 cm, NSCLC in stages III–IV according to the International Union Against Cancer staging system, Karnofsky performance status of ≥70, expected patient survival of over 3 months, progressive disease (PD) after 1 cycle of CCRT, platelet count >20.0 × 10^9^/L, and normal coagulation function. The exclusion criteria were as follows: receiving additional treatment at another hospital; cachexia, systemic metastases, unable to tolerate percutaneous lung biopsy surgery, and severe cardiopulmonary dysfunction.

### Patient Treatment

All of the study patients received 1 initial cycle of CCRT. The commonly used regimens and dosage were as follows: 1000 mg/m^2^ of paclitaxel on day 1, 8, and 15 or 1000 mg/m^2^ of gemcitabine on days 1 and 8, followed by 30 mg/m^2^ of cisplatin on days 1 to 3 or carboplatin at a dose of 300 to 350 mg/m^2^ administered on day 2, and vinorelbine at a dose of 25 mg/m^2^ was administered on days 1 and 8 during radiotherapy every 21 to 28 days. The first course of radiation was given in 2.5 to 3 Gy per fraction, 6 to 7 days a week at a dose of 50 to 70 Gy. As second-line therapy, patients with squamous cell carcinomas received single-agent docetaxel (75 mg/m^2^/3 weeks). Others obtained single-agent pemetrexed (500 mg/m^2^/3 weeks).

### ^125^I seed

The ^125^I seeds type (Yunke Pharmaceutical Limited Liability Company, Chengdu, China) was 6711 to 1985, with a diameter of 0.8 mm, and a length of 4.5 mm. The central source of the particles was a ^125^I radionuclide silver rod, with a diameter of 0.5 mm, and a length of 3.0 mm. The matched peripheral dose (MPD) was 110 to 140 Gy, and the average energy was 27 to 32 KeV. With an initial activity of 0.6 to 0.8 mCi and a half-life of 59.6 days, ^125^I seeds released continuous low-dose γ-ray and soft X-ray (5% of 35 keV and 95% of 28 keV, respectively) after decaying into the organization. The ^125^I seeds had antitumor activity in a radius of 1.7 cm. Within 8 to 10 months, 93% to 97% of the brachytherapy dose was delivered.

### Radiation Dosimetry

**FIGURE 1:**
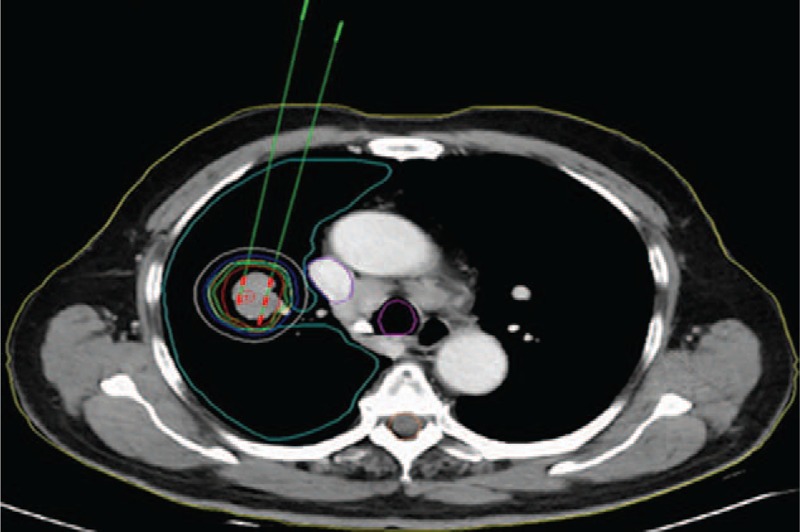
Isodose curves plotted by the TPS. The red line shows the PTV, from inside to outside: the brown, green, orange, blue, and white line were covered by 100%, 90%, 70%, 50%, and 30% of the prescribed dose, respectively. PTV = planned tumor volume, TPS = treatment planning system.

**FIGURE 2:**
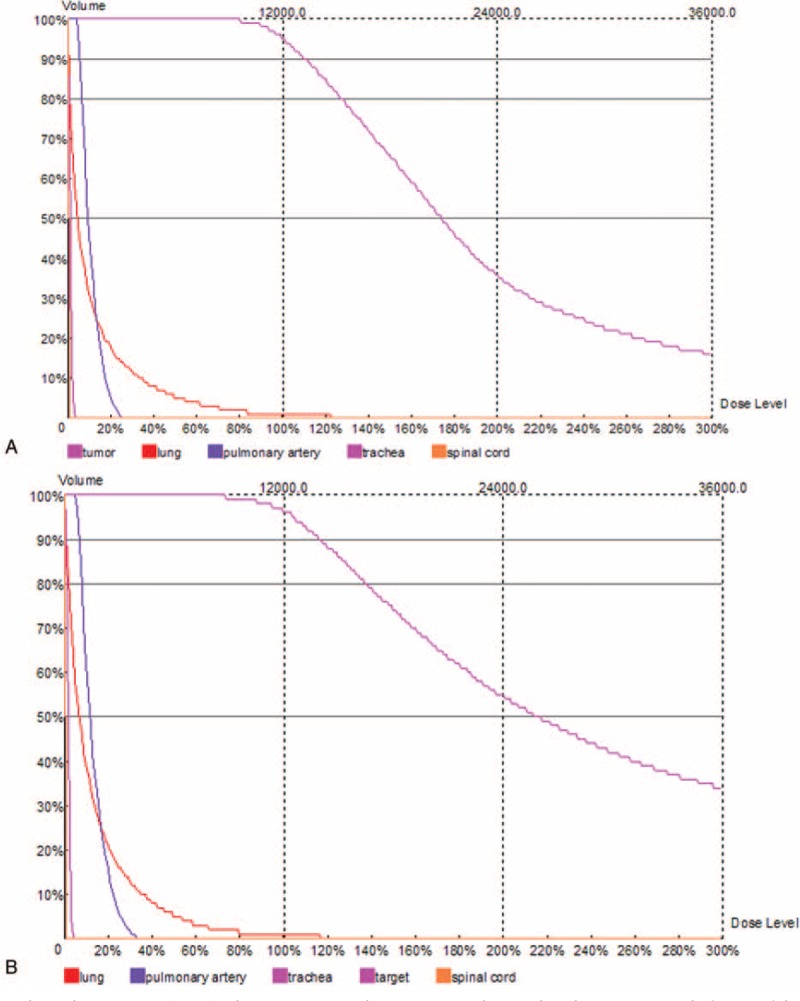
(A) Dose-volume histograms (DVH). The prescription dose is 120 Gy during the planning. A total of 90% of the tumor target (D90) received 132.0 Gy, and 95.4% of the tumor received 100% of the prescribed dose (V100 = 95.4%). D90 was 0, 60 Gy, 0, 0 and V100 were 0.9%, 0, 0, 0 in normal lung, pulmonary, trachea, and spinal cord, respectively. (B) After ^125^I seed implantation, the dose intensity was verified. D90 was 139.2, 1.2, 7.2, 1.2 Gy, 0 and V100 was 96.4%, 0.8%, 0, 0, 0 in tumor, normal lung, trachea, and spinal cord, respectively.

Before ^125^I brachytherapy, an enhanced CT scan, 5-mm thick, was performed a week in all ^125^I seed implantation patients. These images were imported to a treatment planning system (TPS) (RT-RSI, Beijing Atom and High Technique Industries Inc., Beijing, China) to determine the dose of radioactive seeds implanted at the site. A careful delineation of the gross tumor volume (GTV), planning target volume (PTV), and surrounding vital organs was made in every CT slice, PTV was defined as a 1.5 cm of external expansion to the GTV.^21^ The prescribed dose was an averaged 120 Gy (100–140 Gy). Based on the 3 perpendicular diameters within the target tumor and a prescribed MPD of averaged 120 Gy, TPS generated a dose-volume histogram, isodose curves of different percentages, and calculated the dose and number of implanted seeds. PTV edge was covered by isodose curve from 80% to 90% (Figures 1 and 2A). It was necessary to reduce the MPD at important organs and tissues, according to the following criteria: large vessels < 80 Gy, heart 45 to 50 Gy, esophagus < 60 Gy, the spinal cord 45 to 50 Gy, and so on (Figure 2A).

### ^125^I Implantation

Patients were required to sign an operation consent form. They underwent preoperative laboratory examinations and a 2 to 4-hour preoperative fast. Immediately before the procedure, patients were administered a sedative. After sterilization, radiation leak, and activity detection, the ^125^I seed and the one-time implantation gun were sent to the operation room, which was done with caution to prevent radioactive contamination.

**FIGURE 3:**
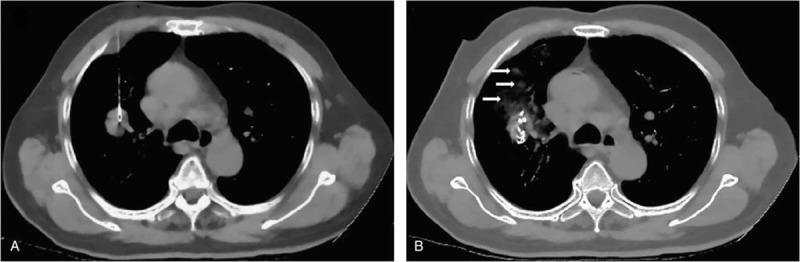
Computed tomography (CT)-guided percutaneous ^125^I seed implantation was performed. (A) The 18 G spinal needle reached the tumor. (B) From deep to shallow, the particles were released, and a small amount of pulmonary hemorrhage was marked by the arrows.

At the beginning of the operation, appropriate positioning was achieved, and the wire was attached to the patient's body surface, marking the puncture position. Then the CT scan, 5-mm thick, was performed to ensure the location and upper and lower borders of the tumor, according to the TPS preoperative plan, with care to avoid the ribs, scapula, major blood vessels, bronchi, and spinal cord. The surgeon determined the direct and shortest puncture level. Local anesthesia was given with 2% lidocaine after disinfection. An 18 G spinal needle reached the farthest tumor edge, but was kept at approximately or less than 5 mm of the border. Similarly, other needles with the same size were penetrated into the tumor, and the distance between every needle was about 1 cm. From deep to shallow, the particles were released while drawing back the needle and keeping adjacent particles at a distance of 10 to 15 mm (Figure 3). To avoid complications during the surgery, all of the spinal needles were retained until the implantation was completed and then removed simultaneously. The CT scan was performed again to exclude any postoperative complications, such as pneumothorax and bleeding. CT scan images were also imported in the TPS program to verify the location of the ^125^I seed implantation and dose intensity (Figure 2B).

### Follow-Up

Those patients who accepted percutaneous ^125^I seed implantation underwent electrocardio monitoring within the following 24 hours. All postoperative symptoms were recorded in detail. Enhanced CT was performed each month for the first 3 months, and then once every 3 months. Follow-up imaging examinations were performed to evaluate the therapeutic effectiveness of ^125^I brachytherapy. All patients were followed up; thus, we were able to observe total response rate (RR), progression-free survival time (PFST), overall survival (OS), and treatment-related complications.

### Evaluation Criteria

The effectiveness of ^125^I brachytherapy was evaluated according to the response evaluation criteria in solid tumors (RECIST). The complete response (CR), partial response (PR), stable disease, and PD were classified accurately. Total RR was the sum of the cases of CR + case of PR)/case number. PFST was defined as the time from the first treatment to the first documented disease progression, death, or end of the study. OS was defined as the time from the first treatment to death of any cause, or to the end of the study. Chemotherapy-related adverse events were evaluated according to the Toxicity Criteria of the World Health Organization (WHO). Adverse effects of irradiation were calculated by the Toxicity Criteria of the Radiation Therapy Oncology Group.

### Statistical Analysis

The statistical software package SPSS version 10.0 was used for statistical analyses. The significance of differences was evaluated using the *t*-test. Response to therapy was analyzed using the Pearson χ^2^ test. Survival analysis was evaluated using the Kaplan–Meier method and the log-rank test. The adverse effect was evaluated using the Wilcoxon rank test. Findings with *P* < 0.05 were considered to indicate a statistically significant difference.

## RESULTS

### Response Rate and PFST

**FIGURE 4:**
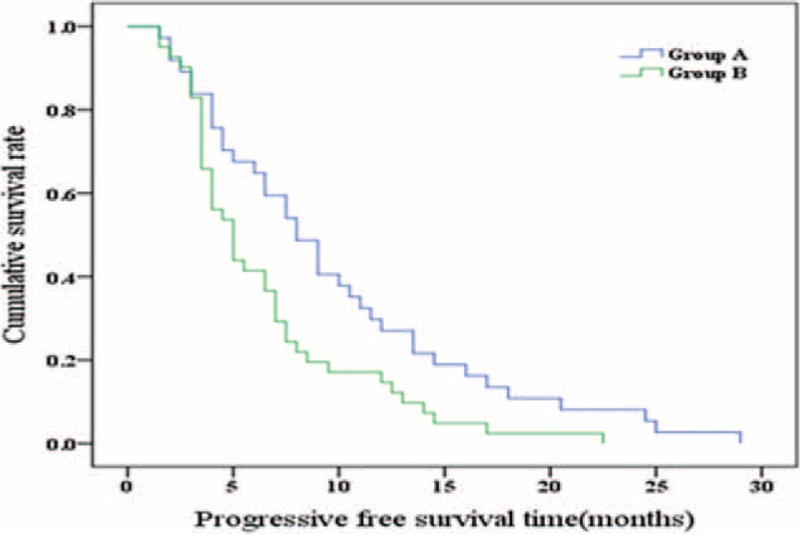
Comparison of progression-free survival time in groups A and B (χ^2^ = 6.392, *P* = 0.011, log-rank test).

The mean follow-up time was 19 (range 3–36) months. In group A, there were 37 patients with 44 lesions, and in group B, there were 41 patients with 41 lesions. After the second treatment in both groups A and B, the CR was 13 (29.5%) versus 1 (2.4%); PR, 15 (34.1%) versus 16 (39.0%); stable disease, 14 (31.8%) versus 17 (41.5%); PD, 2 (4.5%) versus 7 (17.1%); and RR, 28 (63.6%) versus 17 (41.5%). The total RR in tumor response accounted for 63.7% in group A, which was significantly higher than that of group B (41.5%) (*P* = 0.033, Pearson χ^2^ test; Table 2). The median PFST was 8.00 ± 1.09 months (95% confidence interval [CI] 5.87–10.13) in group A and 5.00 ± 0.64 months (95% CI 3.75–6.25) in group B (χ^2^ = 6.392, *P* = 0.011, log-rank test, Figure 4). The difference between groups was statistically significant.

**TABLE 2:**
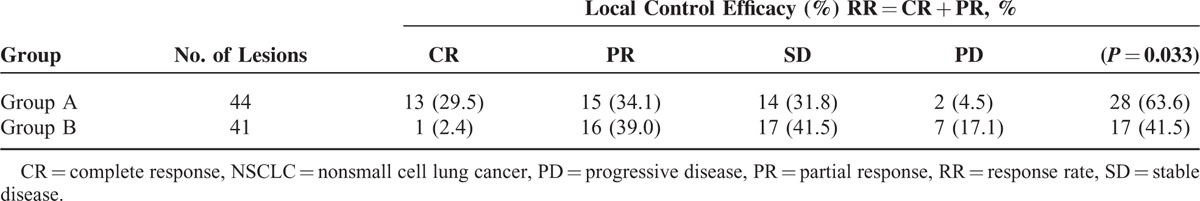
Clinical Efficacy of ^125^I Brachytherapy in Advanced NSCLC

### Overall Survival

**FIGURE 5:**
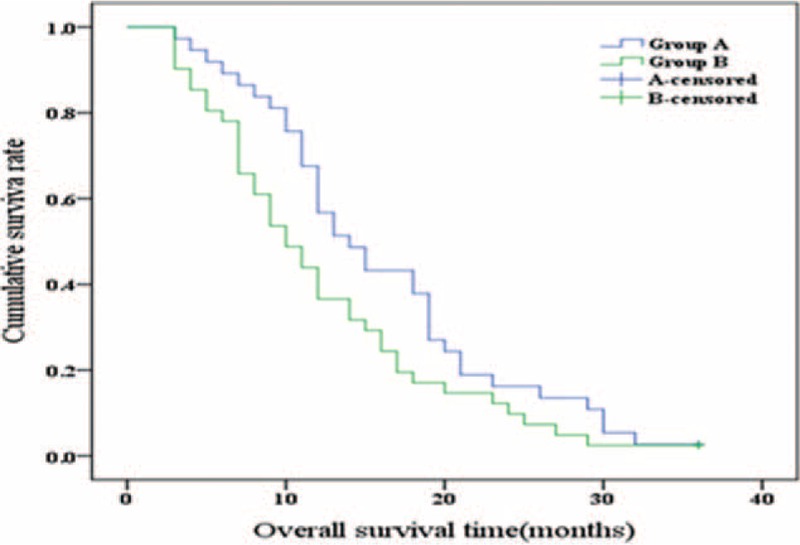
Comparison of the overall survival in groups A and B (χ^2^ = 3.562, *P* = 0.059, log-rank test).

The 1-, 2-, and 3-year OS rates for group A were 56.8%, 16.2%, and 2.7%, respectively, and for group B, 36.6%, 9.8%, and 2.4%, respectively. The median OS time was 14.00 ± 1.82 months (95% CI 10.43–17.58) for the group A and 10.00 ± 1.37 months (95% CI 7.31–12.69) for the group B, respectively (χ^2^ = 3.562, *P* = 0.059, log-rank test, Figure 5). There was no statistically significant difference in survival rates between the 2 groups.

### Interventional- and Radiochemotherapy-Related Complications

Several complications were related to interventional and radiochemotherapy. During the procedure, 3 patients in group A presented pneumothorax with pulmonary compression of more than 30% of the unilateral lung volume and were treated with closed thoracic drainage. The gas was reduced apparently after 2 to 3 days. The remaining 2 patients had no apparent symptoms and did not undergo treatment, because the pneumothorax was less than 30% of the unilateral lung volume. The ^125^I seed was implanted successfully into the tumor in all of the patients in group A. No deaths were caused directly by the treatment given in either group. The toxicities included myelosuppression, gastrointestinal response, fever, allergy, alopecia, radioactive esophagitis, radioactive pneumonia, and local skin reaction. There were no significant differences in terms of complications between the groups (Table 3, *P* > 0.05).

**TABLE 3:**
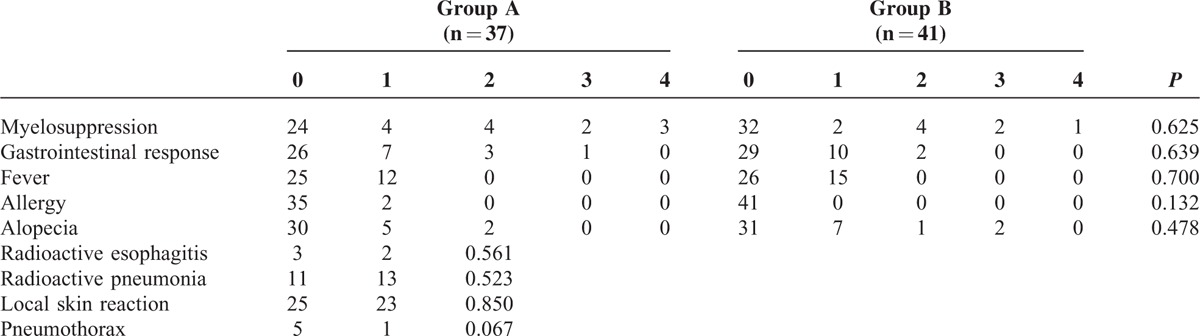
Complications Observed in Groups A and B During Treatment

### Relief of Clinical Symptoms

The common clinical symptoms of patients with stage III–IV NSCLC were cough, chest pain, bloody sputum, hoarseness, and chest tightness. We found changes in these clinical symptoms by comparing both groups. The relief of symptoms associated with the tumor in group A was better than that in group B (Table 4, *P* < 0.05). We found significant remission in 20 patients with cough, and 17 with chest pain in group A. In group B, 5 had cough and 2 had chest pain. In group A, 9 patients with cough and 7 with chest pain had partial response, as well as 4 patients with cough and 5 with chest pain in group B. Similarly, the rates of remission (significantly + partial) of bloody sputum, hoarseness, and chest tightness were 13/16, 5/6, 9/11 and 6/20, 2/9, 4/15, respectively.

**TABLE 4:**
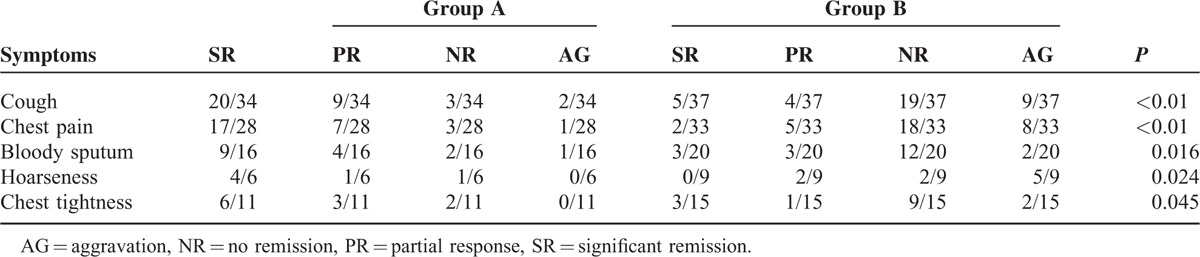
Relief of Symptoms Associated With the Tumor in Both Groups

## DISCUSSION

For locally advanced NSCLC patients who have not received any treatments, the excepted survive time is less than 6 months and the effect of surgical treatment has also been debated.^22,23^ The goal of treatment for stages III–IV NSCLC is local control and the prevention of systemic metastases.^24^ The incidence of systemic metastasis was reduced, and the survival time of patients was prolonged by the remission of local tumor. Radiotherapy, especially CCRT, currently plays a major role in the management of lung cancer. The reason is that most patients are not surgical candidates because of the advanced disease stage at diagnosis, poor clinical condition, and comorbidities.^25^ O’Rourke et al^26^ reported that CCRT is the standard of care in local advanced NSCLC with a median survival rate of approximately 21 months. McCloskey et al^27^ found that CCRT decreases loco-regional progression (hazard ratio [HR] = 0.77; *P* = 0.01), which translates into a significant survival benefit (HR, 0.84; 95% CI, 0.74–0.95; *P* = 0.004). The absolute survival benefit was 5.7% at 3 years and 4.5% at 5 years. However, at present, because patients had poor physical health, severe toxicity after CCRT, or complications with other serious diseases, more than half of the patients were unable to tolerate the standard treatment modality and continue with the subsequent treatments.^28,29^ These factors resulted in locally advanced NSCLC recurrence and distant metastasis.

It is necessary that the radiation dose reaches a certain level to destroy tumor cells in intrathoracic malignant tumors. The lowest dose that can control tumor progression is 80 to 120 Gy.^30^ Many studies have certified that increasing the radiation dose significantly improved local control rate. Perez et al^31^ reported that after doses of 40, 50, and 60 Gy were applied to NSCLC patients, 2-year survival rates were 11%, 19%, 19%, respectively. Dosoretz et al^32^ conducted a study in which the doses >70, 61 to 70, 50 to 60, and <50 Gy of radiotherapy were given to patients with unresectable NSCLC stages I–III, and the 2-year survival rates were 50%, 33%, 22%, 0, respectively. However, because of normal tissue damage and long-term external irradiation, especially for tumor tissue involving vital organs (heart, great vessels, trachea, esophagus, and spine), significantly less radiation doses are administered to the tumor tissue.^33^ Even with the use of advanced technology, including the development of image guided radiotherapy (IGRT), stereotactic ablative body radiotherapy (SABR), and intensity modulated radiotherapy (IMRT), the lowest effective dose could not be reached, leading to treatment failure.

Radioactive ^125^I seed implantation treatment is internal radiotherapy, and it has more obvious advantages than external beam radiotherapy. First, the localized radiation dose of the target is higher, but lower for the surrounding normal tissues.^34^ Second, persistent low-dose irradiation could affect the different cell cycle stages, so it can continue to kill tumor cells.^35^ Third, when killing tumor cells, the rays have lower oxygen dependence, and this can improve the killing effect on tumor cells.^36^ Last, it increases the relative biological effect.^37^ In the present study, the ^125^I particle combined with the preoperative TPS allows the PTV to cover more than 95% of the tumor volume, which will receive 100% of the radiation dose. Therefore, we could guarantee the administration of a sufficiently high dose to the local tumor without damaging the surrounding tissue.

Brachytherapy has been widely used in prostate cancer, liver cancer, and brain tumors, with good results. Yorozu et al^38^ reported that a permanent ^125^I seed implantation treatment of prostate cancer is safe and efficient. Nag et al^39^ found that ^125^I brachytherapy was a safe and effective alternative to other locally ablative techniques, and that it can provide long-term local control. The overall 1-, 3-, and 5-year actuarial intrahepatic local control rates were 44%, 22%, and 22%, respectively. Huang et al^40^ also reported that excellent local control was achieved using permanent ^125^I brachytherapy for brain metastasis resection cavities. But it has few studies about locally advanced NSCLC, in our study. The total RR was 63.6% in the group A and 41.5% in the group B (*P* = 0.033). It further proves that ^125^I seed implantation is feasible and safe in lung malignancies.^41,42^

Locally advanced NSCLC, especially central lung carcinoma, could easily invade the pulmonary arteries and veins, and trachea.^43^ During implantation, the particles may be implanted to the pulmonary arteries or trachea causing pulmonary embolism or obstructive atelectasis. Hence, during the puncture process of every central lung cancer, we performed an arterial enhanced CT scan. None of the patients in our study experienced pulmonary embolism or atelectasis caused by the particles of implantation. Thus, this finding implies that ^125^I seed implantation was safe and practical.

Radiation pneumonitis and esophagitis were the most common complications. We observed similar chemotherapy toxicities (bone marrow suppression, gastrointestinal reactions, and fever) in both groups. The radiation pneumonitis and esophagitis, respectively, in group A were 11 and 3 cases, which were 13 and 2 cases in group B; there was no statistically significant difference between the 2 groups. After the appropriate treatment was administered, the condition improved significantly. This finding indicated that there was a very little effect on the body by ^125^I implantation; it did not represent additional risks for patients than CCRT. This was consistent with previous studies.^44–46^ Additionally, group A showed no surgery-related lethal hemoptysis, esophagotracheal fistula, or life-threatening morbidity. There were 5 patients with pneumothorax after implantation. One of them amounted to >50% of the unilateral lung volume, which resulted in chest tightness, cardiac pain, and dyspnea. Two cases with 30% to 50% pneumothorax had mild chest discomfort. These patients showed improvement after immediate closed thoracic drainage. The remaining 2 patients with <30% pneumothorax were asymptomatic and were not given any specific treatment. These cases resolved completely after 24 hours as shown by the repeat chest radiograph taken. After reviewing the data, we found out that pneumothorax possibly resulted from tumor location, pulmonary function, biopsy needle diameter, rib blockage, and breathing mechanics during the operation.^47,48^ First, due to more lung tissue was passed during the therapy, the incidence of pneumothorax was significantly higher in central lung cancer than in peripheral lung cancer. Second, patients with chronic obstructive pulmonary disease and emphysema, especially bulla, had an increased incidence of pneumothorax. Third, because rib blockage and breathing mechanics made the direction changes of the needle during insertion, they may lead to repeated puncture, which increased lung injury risks obviously. Lastly, the pneumothorax was also more obvious by using the larger diameter of needle. Thus, we should assess comprehensively the patient's conditions before ^125^I seed implantation so that tried to avoid complications (such pneumothorax) by selecting appropriate patients.

**FIGURE 6:**
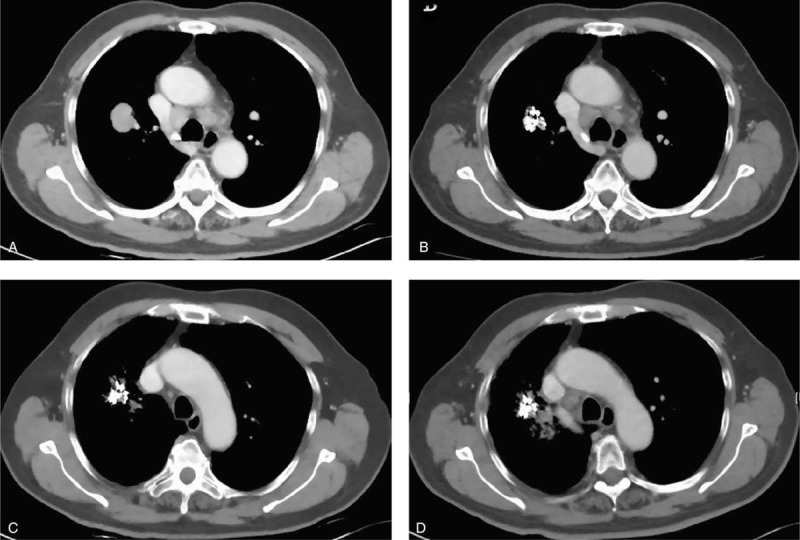
A 70-year-old male patient. (A) CT scan before treatment, (B–D) 1, 2, and 3 months after ^125^I implantation treatment, the lesion apparently shrunk (CR). The particles gathered and did not shift to the normal lung. CT = computed tomography, CR = complete response.

Treatment goals for patients with stage III–IV NSCLC were not only improving local response and prolonging life, but also palliating symptoms and improving quality of life.^49^ During the postoperative follow-up, we found significant improvement in the relief of symptoms associated with tumors in group A. Over 50% of patients with advanced lung cancer experience cough and chest pain symptoms. The most effective current treatment for them was comprehensive cancer treatment. Cough and chest pain remission rates in the 2 groups (significantly remission + partly remission) were 29/34, 24/28 and 9/37, 7/33, respectively (*P* < 0.01). Although ^125^I seed implantation remission cough and chest pain mechanisms are not fully understood, radiation therapy that shrinks tumors or inhibits tumor cells was seen as the main reason. However, we found that tumor shrinkage does not fully explain the cough and pain relief. In our study, 1 patient with peripheral lung cancer had a constant complaint of chest discomfort and cough, although his CT scan 1 month after the implantation showed significant reduction in tumor size. It may be due to accumulation of the particles closer to the pleura, which has higher sensitivity to radiation. Such symptoms may also be caused by the difference in individual tolerance. This may require further clinical and statistical exploration. After the local tumor decreased in size, the particles gathered along the needle tract and did not shift to the normal lung tissue (Figure 6). After gathering, the particles were closer to important tissue, such as blood vessels, heart, and esophagus. However, no radiation-related complications were found in these patients.

Previous studies had found that the incidence of bloody sputum after seed implantation was 0% to 20%.^50^ In group A, postoperative bloody sputum was worse than that in preoperation in 1 patient. The CT scan also confirmed the bleeding inside the pulmonary lesions. The patient was advised to avoid strenuous coughing and given hemostatic drugs for 3 days. There was significant improvement in 3 to 5 days. Hemoptysis may be caused by simultaneous penetration of the trachea and blood vessels by the needle during implantation or by repeated puncture. This may need further clinical studies.

In conclusion, CT-guided ^125^I seed implantation proved to be a potential approach in treating localized advanced NSCLC. It provided good local control and relieved clinical symptoms without increasing side effects. But our study was limited by its retrospective design, sample size, and relatively short follow-up period for some patients. Future clinical trials need to focus on the role of the treatment of local and systemic treatment for locally advanced NSCLC to improve local control and quality of life. Finally, more prospective studies should be carried out.
